# The additional role of virtual to traditional dissection in teaching anatomy: a randomised controlled trial

**DOI:** 10.1007/s00276-020-02551-2

**Published:** 2020-09-17

**Authors:** Rafael Boscolo-Berto, Cinzia Tortorella, Andrea Porzionato, Carla Stecco, Edgardo Enrico Edoardo Picardi, Veronica Macchi, Raffaele De Caro

**Affiliations:** 1 Department of Neurosciences, Institute of Human Anatomy, University of Padova, Via Gabelli 65, Padova, Italy; 2 Veneto Region Reference Center for the Preservation and Use of Gifted Corpses, Veneto Region, Via Gabelli 65, Padova, Italy; 3 Digestive System Surgery Division, European Institute of Oncology (IRCSS), Via Ripamonti 435, Milano, Italy; 4 Vesalio Center for Anatomical Training “A Vesalius”, University of Padova, Via Gabelli 65, Padova, Italy

**Keywords:** Body donation, Cadaver dissection, Clinical anatomy, Education, Randomised controlled trial, Virtual dissection

## Abstract

**Introduction:**

Anatomy has traditionally been taught via dissection and didactic lectures. The rising prevalence of informatics plays an increasingly important role in medical education. It is hypothesized that virtual dissection can express added value to the traditional one.

**Methods:**

Second-year medical students were randomised to study anatomical structures by virtual dissection (intervention) or textbooks (controls), according to the CONSORT guidelines. Subsequently, they applied to the corresponding gross dissection, with a final test on their anatomical knowledge. Univariate analysis and multivariable binary logistic regression were performed.

**Results:**

The rate of completed tests was 76.7%. Better overall test performance was detected for the group that applied to the virtual dissection (OR 3.75 with 95% CI 0.91–15.49; *p* = 0.06). A comparable performance between groups in basic anatomical knowledge (*p* 0.45 to 0.92) but not muscles and 2D-3D reporting of anatomical structures was found, for which the virtual dissection was of tendential benefit (*p* 0.08 to 0.13). Medical students who applied to the virtual dissection were over three times more likely to report a positive outcome at the post-dissection test than those who applied to textbooks of topographical anatomy. This would be of benefit with particular reference to the understanding of 2D–3D spatial relationships between anatomical structures.

**Conclusion:**

The combination of virtual to traditional gross dissection resulted in a significant improvement of second-year medical students’ learning outcomes. It could be of help in maximizing the impact of practical dissection, overcoming the contraction of economic resources, and the shortage of available bodies.

**Electronic supplementary material:**

The online version of this article (10.1007/s00276-020-02551-2) contains supplementary material, which is available to authorized users.

## Introduction

Human gross anatomy represents a pillar of medical curricula worldwide and is aimed at introducing the core anatomical concepts within the clinical context.

From a historical point of view, the study of anatomy by dissecting cadavers has been the most relevant teaching paradigm for human gross anatomy learning, and in our institution can count on a body donation program for anatomical education implemented since the 2000s [9].

Nevertheless, given the exponential development of interest in anatomical exercise on cadavers by students, post-graduates, and specialists, together with the increase of class sizes, the number of bodies available is still insufficient to meet needs. As a result, the quality of dissection over the number of cadavers dissected became stressed.

However, there is an endless debate on the useful balance between the advantages of dissections against the difficulties in cost, availability of bodies, and facility management of dissection theatres [3]. The paradigm shift has moved from passive and teacher-centred learning towards active and student-centred learning, hence making the sole combination between lectures and learning based on traditional textbooks inadequate for the emerging integration of multi-modal teaching resources. Innovation includes, among the others, interactive three-dimensional techniques showing the potential to improve the spatial knowledge of anatomy [28]. Cross-sectional and three-dimensional visualization of anatomical structures (i.e., virtual dissection) seemed to be effective for the retaining of anatomical details and the understanding of their neighbourhood relationships [19]. Moreover, it has demonstrated a favourable impact on student’s learning and perception [8].

Hence, in an attempt to overcome the above-mentioned limitations in teaching anatomy, virtual dissection was identified as a potentially useful option to allow the emulation of anatomical dissection as close as possible to reality, notwithstanding the physical absence of a corpse. However, it is unclear what the resulting consensus is on whether or not to integrate technological innovations into gross anatomy courses. Over time, scientific evidence has accumulated in favor of emerging technologies [7, 12–14, 21, 22, 24, 25] and against these [10, 15–17, 20]. As a consequence, modern medical curricula do not systematically incorporate these novel learning tools yet.

As such, the present study hypothesized that integrating the classical gross dissection with a supplemental virtual experience on digital human cadaver can improve the learning of anatomy with benefits on students’ performance, particularly concerning the three-dimensional representation of relationships between anatomical structures in a specific district, hence maximizing the impact of practical experience.

## Methods

**Fig. 1:**
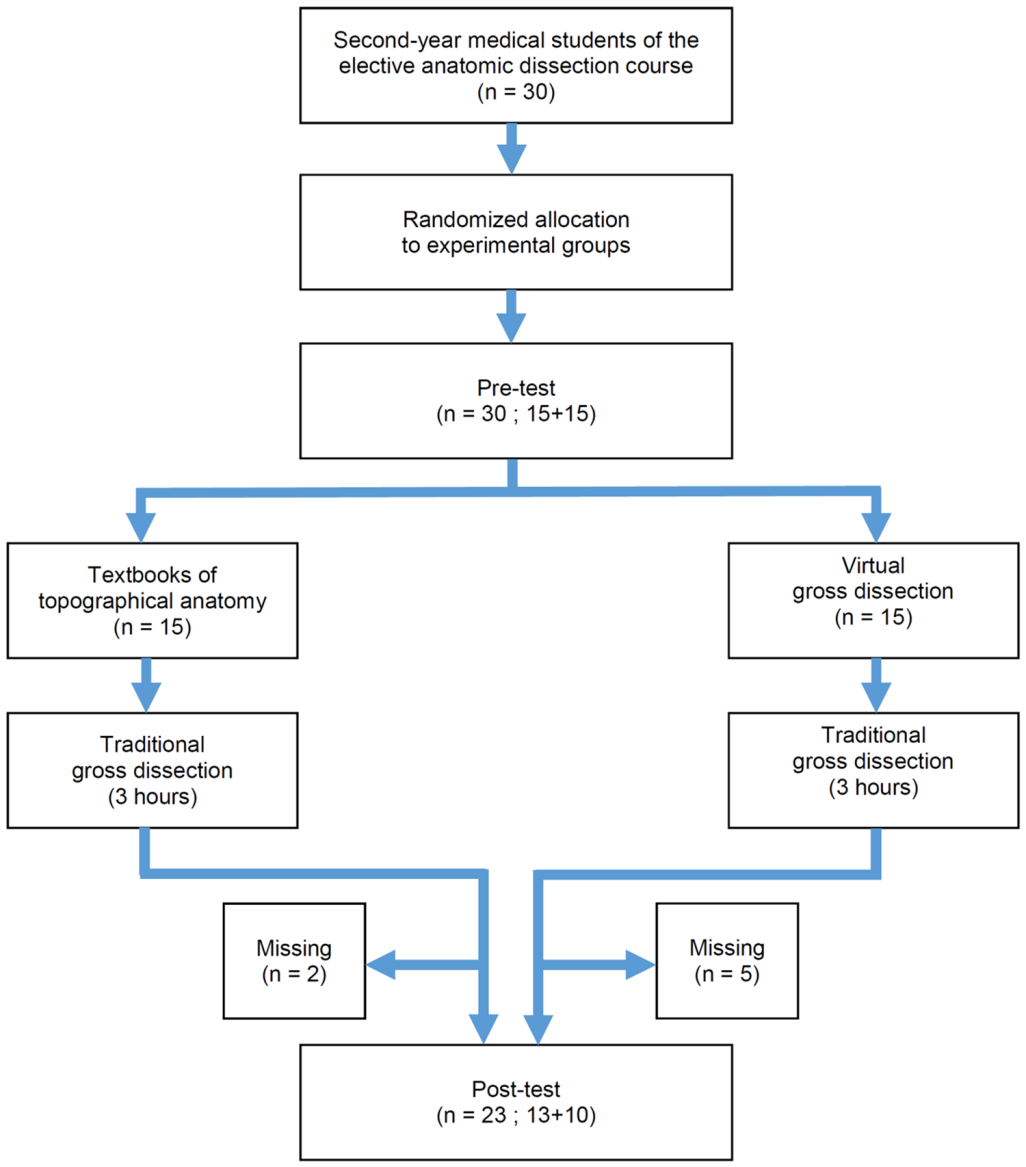
Randomised controlled didactical trial flow-chart

A randomised controlled didactical trial was implemented to test the hypothesis and verify the usefulness in teaching anatomy of virtual dissection added to the classical approach by gross dissection on the human fresh frozen body (Fig. 1).

The CONSORT guidelines and checklist for randomised trials were followed (Supplementary material). The primary outcome consisted of the mean variation of the final overall score obtained at the post-experimental examination. Secondary outcomes consisted of the mean variations of the subscores regarding the listing, the 2D and 3D reporting of anatomical structures.

### Participants, randomisation to groups and variables

One author (BBR) enrolled participants, managed the allocation, and assigned participants to the sequence of interventions.

All the 30 second-year medical students who took part in the elective anatomic dissection course during the gross anatomy course were considered eligible. As the number of applications was equal to the number of available places, a selection test was not carried out. Moreover, as the trial had a limited number of students available, the preliminary calculation of the sample size was not carried out. Enrolled students were 18 males and 12 females. The mean age of participants was 20.9 ± 0.6 years, ranging from 20.1 to 22.1 years. All medical students were unpaid volunteers, and none of them had any previous experience of forearm dissection.

Student’s personal data on their academic career (variables: ‘scoring arithmetic mean for passed examinations’ (SAM), ‘scoring weighted mean for passed examinations’ (SWM), ‘scoring obtained at the anatomy examination’ (SAE), ‘number of repeated examinations’ (NRE), ‘ranking in the national selection test’ for admission to medical school (CNS)) and their anatomical knowledge (‘scoring obtained at the preliminary test’ (SPT)) were collected. They were subsequently randomised to a first group (‘Virtual Dissection Group’), which applied for 20 min to a virtual dissection aimed at learning anatomy, and a second one applied for 20 min to textbooks of topographical anatomy (‘Textbook Group’). Randomisation was performed on the Random-Order-of-Service (ROS) basis, and the allocation ratio was 1:1. Moreover, a certain similarity of the experimental interventions was implicit because the reference was to the same anatomical region. A significant part of anatomical learning is related to the integration of the systematic information learned in a three-dimensional mental representation that synthesizes the different relationships between structures present in a specific anatomical district. This processing allows us to mature an anatomical knowledge adhering to the real three-dimensionality of the human body with which the physician will have to interact. For these reasons, the anatomical topic of interest was stated as being the forearm (bones, muscles, vessels, and nerves). The type of experiment prevented the blinding process. The trial took a week to complete.

### Pre-test

At the beginning of the elective course, students were asked to complete a non-announced examination with ten multiple-choice questions (Supplementary material—Pre-test), they had 30 min to answer.

All questions were related to topographical anatomy, and questions were phrased purely using five text sentences each. All questions either consisted of statements whose correctness had to be evaluated or of positively and negatively formulated statements with only one of them being correct. There were five multiple-choice options to answer. All questions counted equally, such that the maximum number of achievable points was 10.

#### Textbook consultation (referred to the textbook group—controls)

Textbooks of topographical anatomy were provided to the students in a comfortable setting for the expected time.

#### Virtual dissection (referred to the virtual dissection group—interventions)

A unique context for studying anatomy by additional virtual dissection was provided by using of one life-size virtual dissection table at the Institute of Anatomy of the University of Padova. The Anatomage Table (Anatomage Inc., San Jose, Ca, USA) is a hands-on three-dimensional medical education tool that enables students to dissect a digital human cadaver virtually. It presents an interactive touch display measuring 2.1 m × 0.6 m, onto which students can manipulate structures in any plane or slice in cross-section, create 3D reconstructions at multiple angles, and label the selected anatomy [29]. The system had preinstalled whole body cross-sections of fresh frozen cadaver specimens.

### Gross dissection

Following this preliminary step, the students applied to the three-hour gross dissection of a human forearm, conducted on a fresh frozen body of an adult male of the Body Donation Program ‘Donation to Science’ of the University of Padova, Italy [9]. The scientific coordinator of the donation program at the University of Padova approved the study.

A post-dissection test aimed to evaluate their retained information with regard to 2D and 3D anatomical structures other than basic anatomical knowledge was administered. It provided a score which was coded as positive (‘passed exam' (PE)) if it was greater than 70% of the maximum score obtainable.

### Post-test

The questions of the post-test were categorized into the learning taxonomy of Bloom. The test featured four questions from two taxonomic levels of difficulty distributed equally as either “Knowledge” and “Comprehension” (Supplementary material—Post-test). For the former, students were asked to retrieve, recognize, and recall relevant knowledge from memory. Information assessed were basic definitions and terms to be listed. The latter means that students were asked to demonstrate a basic understanding of 2D and 3D spatial relationships referred to bones, muscles, vessels, nerves of both proximal and distal forearm sections, by self-drawing the pictures of required sections. Information assessed was the topographical relationships between anatomical structures in a given district. The test was administered immediately after the planned traditional gross dissection. Students had 30 min to answer all questions. The examination was considered as passed if the test provided more than 70% of the maximum score obtainable.

The reliability of the post-test was good regarding the test using Cronbach’s alpha (*α* = 0.84). The inter-rater reliability of the post-test was assessed by Cohen’s kappa and resulted substantial regarding the test (*k* = 0.78).

Free expression of one's thought has been asked in the form of a single written sentence to qualitatively assess the students’ and teachers’ opinions on virtual dissection.

### Statistical analysis

The parametric nature of studied variables was assessed by measures of central tendency, measures of variability, and measures of shape, and confirmed by performing of Shapiro–Wilk test. As a consequence, parametric variables were described as mean ± standard deviation, while non-parametric variables were reported as median with interquartile range (IQR = Q3−Q1). The comparison between variables in univariate analysis was performed by the *t*-Student test or Mann–Whitney *U*-test, as appropriate.

For the regression calculation, a preliminary check for correlations between independent and the dependent variables, for correlation between independent variables, as well as for multicollinearity was performed. A multivariable binary logistic regression model was performed with the ‘passed exam’ as an outcome (passed/positive as reference) and the others as potential independent variables (explanatory variables), to assess their impact on the likelihood that a student passes the post-dissection test. This was done by using the Wald chi-squared test, which allows to find out if explanatory variables in a model are significant, i.e. they add something to the model, or add nothing and can be deleted without affecting the model itself.

A two-sided *p* < 0.05 was chosen to indicate statistical significance in all the analyses, whilst *p*-levels between 0.05 and 0.10 were considered tendentially significant and clearly stated in the paper. Whenever a statistical result did not reach the statistical significance with a *p*-value close to the significant level, a power analysis was performed to quantify the effective probability of rejecting a false null hypothesis.

Statistical tests were performed by IBM Statistical Package for the Social Sciences (SPSS), Version 17.0 (Chicago, SPSS Inc.), and Piface software by R.V. Lenth, Version 1.76.

## Results

The rate of completed tests was 76.7% (23/30). Of the missing (5 females and 2 males), 5 out of 7 were into the Virtual Dissection Group (resulting *n* = 10), the others into the Textbook Group (resulting *n* = 13). They did not carry out the post-dissection test due to factors independent of the study design, the administered test, or their knowledge of anatomy.

Preliminary comparisons of considered variables between subgroups (Virtual Dissection vs Textbook Groups) verified the effectiveness of random allocation (Table 1), as no statistical differences were detected among groups, with *p*-values ranging from 0.23 to 0.97.

**Table 1:** Descriptive statistics

Variable	Overall (*n* = 23)	Virtual dissection group (*n* = 10)	Textbook group (*n* = 13)	*p*
Age (years)	20.9 ± 0.6	21.0 ± 0.6	20.8 ± 0.4	0.23
Gender				0.97
Males	16 (70%)	7 (70%)	9 (69%)	
Females	7 (30%)	3 (30%)	4 (31%)	
Scoring arithmetic mean for passed examinations	26.9 ± 1.7	27.1 ± 2.1	26.7 ± 1.5	0.55
Scoring weighted mean for passed examinations	26.7 ± 2.0	26.9 ± 2.4	26.6 ± 1.6	0.70
Scoring obtained at the anatomy examination	25.3 ± 1.7	25.6 ± 2.4	25.2 ± 2.1	0.74
Number of repeated examinations	1.2 ± 1.6	0 [1]	1 [2]	0.27
Classification in the national selection test	1418 ± 938	1332 ± 643	1483 ± 1137	0.71
Scoring obtained at the preliminary test				0.65
Absolute score	5 ± 1.7	5 [1]	5 [2]	
Relative score (%)	50 ± 17	50 [10]	50 [20]	
Scoring obtained at the post-test				0.62
Absolute score	10.9 ± 3.2	11.3 ± 3.7	10.6 ± 2.9	
Relative score (%)	68.2 ± 20.1	70.6 ± 23.4	66.3 ± 18.0	
Participants passing the examination (*n*)*	11 (47.8%)	5 (50%)	6 (46.1%)	

Parametric variables were described as mean ± standard deviation; non parametric variables are reported as median with interquartile range (Q3−Q1) within square brackets

Preliminary comparisons of considered variables between virtual dissection vs textbook groups were performed by *t*-Student test or Mann–Whitney *U*-test as appropriate, to verify the effectiveness of random allocation. A two-sided *p* < 0.05 indicated statistical significance

*The final post-experimental test provided a score which was coded as positive ['passed examination' (PE)] if it was greater than 70% of the maximum score obtainable

The scoring obtained at the post-test showed a better performance for the Virtual Dissection Group, even if not statistically significant under a low power comparison (70.6 ± 23.4 vs 66.3 ± 18.0; *p* = 0.62; power 10%).

On univariate analysis (Table 2), medical students passing the post-dissection test showed a higher scoring obtained at the previous anatomy examination (27 with IQR 2.2 versus 24.6 with IQR 2.1; *p* < 0.05). None of the other variables (‘scoring arithmetic mean for passed examinations’, ‘scoring weighted mean for passed examinations’, ‘number of repeated examinations’, ‘ranking in the national selection test’, ‘scoring obtained at the preliminary test’ and ‘experimental group allocation’) showed a significant statistical difference between the Passed Examination Group and the Not passed Examination Group (*p* ranging from 0.11 to 0.82). Nevertheless, to quantify the effective probability of rejecting a false null hypothesis with *p*-values close to the significant level, a power analysis was additionally performed, detecting statistical values overall ranging below 41%. As a consequence, considering that the selection process of variables based on univariate analysis for the subsequent multivariable analysis is just a pre-selection strategy and no inference will derive from this step, the introduction of all the variables into the regression modeling without reducing their initial number was chosen, hence reducing the risk of missing important variables.

**Table 2:** Univariate analysis

Variable	Overall (*n* = 23)	Passed examination group (*n* = 9)*	Not passed Examination group (*n* = 14)*	*p*
Scoring arithmetic mean for passed examinations	26.9 ± 1.7	27.9 [2.3]	26.4 [2.7]	0.13
Scoring weighted mean for passed examinations	26.7 ± 2.0	28.1 [2.4]	26.2 [3.5]	0.11
Scoring obtained at the anatomy examination	25.3 ± 1.7	27 [2.2]	24.6 [2.1]	< 0.05
Number of repeated examinations	1.2 ± 1.6	1 [2]	1 [2]	0.62
Ranking in the national selection test	1418 ± 938	929 [1183]	1843 [1306]	0.19
Scoring obtained at the preliminary test				
Absolute score	5 ± 1.7	5 [2]	5 [2]	0.82
Relative score (%)	50 ± 17	50 [20]	50 [20]	
Experimental group allocation (*n*)				
Virtual dissection group		5	5	
Textbook group		4	9	0.36

*The final post-experimental test provided a score which was coded as positive ('passed examination' (PE)) if it was greater than 70% of the maximum score obtainable. Parametric variables were described as mean ± standard deviation; non parametric variables are reported as median with interquartile range (Q3−Q1) within square brackets. Univariate analysis between virtual dissection vs textbook groups was performed by *t*-Student test or Mann–Whitney *U*-test as appropriate for normality. A two-sided *p* < 0.05 indicated statistical significance

The preliminary checking for interfering and unacceptable correlations or multicollinearity leads to the discard of variables ‘number of repeated examinations’, ‘scoring weighted mean for passed examinations’, and ‘ranking in the national selection test’. The reduced model on logistic regression containing the selected predictors for a positive outcome at the post-dissection test (‘scoring arithmetic mean for passed examinations’, ‘scoring obtained at the anatomy exam’, ‘scoring obtained at the preliminary test’, ‘experimental group allocation’) was statistically significant (*χ*^2^ = 9.39, *p* = 0.05), indicating that the model was able to distinguish between the ‘passed/positive’ and ‘not passed/negative’ outcome. The model as a whole explained between 18.5% (Cox and Snell *R* square) and 25.0% (Nagelkerke *R* squared) of the variance for the 'passed exam', and correctly classified 78.3% of cases.

As shown in Table 3, only the variable ‘experimental group allocation’ made a unique statistically significant contribution to the model (OR 3.75 with 95% CI 0.91–15.49; *p* = 0.06). However, as in this setting, the logistic regression of the binary response variable ‘passed exam’ on ‘experimental group allocation’ between Virtual Dissection Group or Textbook Group achieves 28% power at a 0.05 significance level to detect as significant a change corresponding to an odds ratio of 3.75; the *p*-value (0.06) was interpreted as tendentially significant, hence deserving full consideration.

**Table 3:** Binary logistic regression predicting likelihood of passing the post-experimental test

	B	S.E	Wald	*p* value*	Odds ratio	95% C.I. for odds ratio	
						Lower	Upper
Scoring arithmetic mean for passed examinations	0.279	0.262	1.140	0.28	1.322	0.792	2.208
Scoring obtained at the anatomy examination	0.173	0.184	0.881	0.34	1.189	0.829	1.705
Scoring obtained at the preliminary test	− 0.155	0.262	0.351	0.55	0.856	0.513	1.430
Experimental group allocation Virtual dissection group Textbook group	1.320	0.724	3.326	**0.06**	3.745	0.906	15.478

*B* intercept in the null model, *S.E.* standard error, *C.I.* confidence interval

A two-sided *p* < 0.05 indicated statistical significance, whilst *p*-levels between 0.05 and 0.10 were considered tendentially significant

*Numbers in bold are referred to as variable included into the final model

This indicated that medical students who applied to a virtual dissection aimed at learning anatomy were over three times more likely to report a positive outcome (passed/positive) at the post-dissection test than those who applied to textbooks of topographical anatomy, controlling for all other factors in the model.

**Fig. 2:**
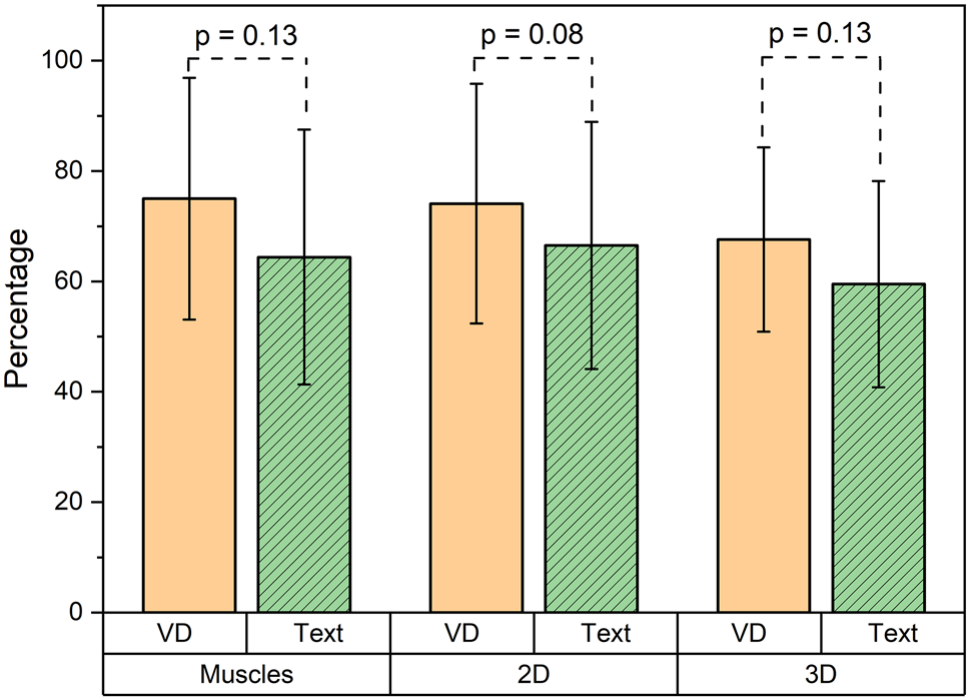
Focus on the subset of tendentially significant subscores comparisons regarding the listing, the 2D and 3D reporting of anatomical structures. Text: experimental group randomised to the use of textbooks of topographical anatomy; VD: experimental group randomised to the virtual dissection; 2D: reporting of anatomical structures organized on a bidimensional plane; 3D: reporting of anatomical structures organized on a tridimensional volume. Percentages are referred to the proportion of correct answers provided

Although further deepen the analysis of secondary outcomes was prevented due to the under-powering of the study, additional data may be provided (Fig. 2 and Fig. 3 provided as Supplementary material). Even if there were no differences between the two groups regarding the listing of vessels, nerves, and bones (*p* 0.45 to 0.92), the correct percentage in listing the muscles seemed to show a better performance for the Virtual Dissection Group, although not statistically significant (75 ± 21.9 vs 64.4 ± 23.1; *p* = 0.13). Similar findings underscoring the virtual dissection benefit, were noted for the percentages of correct results in 2D reporting of anatomical structures (74.1 ± 21.7 vs 66.5 ± 22.4; *p* = 0.08); and 3D reporting of overall anatomical structures (67.6 ± 16.7 vs 59.5 ± 18.7; *p* = 0.13).

Overall, 70% of all the students belonging to the group who used the virtual dissection reported that the use of virtual dissection was beneficial to their learning—especially for three-dimensional expectations—and more engaging experience than studying textbooks and atlases. From a qualitative point of view, the students’ opinion on virtual dissection was reckoned, showing a fair interest in virtual dissection they tested, appreciating the possibility of enlarging the anatomical details. However, difficulties in the first approach to the instrument were reported, even being overcome during the first few minutes of practice. Subsequently, the possibility of exploration of the districts was generally appreciated, even if the teachers occasionally noted some off-task behaviours. Some complaints were expressed for occasional table slowing, images with poor details at the highest zooming, limitations to the default manipulation, and the restriction of interaction to one person at a time. Generally, the traditional gross dissection of a real body was still unanimously reported as the pivotal approach to the learning anatomy, judged superior to any potential virtual substitute. Anyhow, the preliminary usefulness of virtual dissection for subsequent application to traditional cadaveric dissection was recognized.

## Discussion

A solid understanding of gross human anatomy is recognized as a vital element to the medical curriculum and essential in all medical specialties, as accurate diagnosis of alterations in human organs or systems requires a deep preliminary knowledge of normal human anatomy.

The dissection of cadavers and didactic lectures have been a key point of teaching anatomy since the Renaissance. However, increasing student enrolments jointly to simultaneous decreasing numbers of cadavers have made it more difficult for medical students to access and dissect cadavers within their more and more limited laboratory training time and reduction of hours devoted to cadaveric-based teaching. This general trend is culminating, especially among European schools, into the disappearing of the discipline from many academic institutions [19, 26]. Hence, the teaching of anatomy is now experiencing its most profound crisis. At the same time, today’s medical students are paradoxically challenged by the quantity and complexity of the anatomical structures they have to learn, with a significant risk of compromised anatomical knowledge and, subsequently, the standard of healthcare in all the medical disciplines.

In this scenario, the rising prevalence of informatics might play an increasing role in medical education, potentially representing an integrative system to be combined with the dissection of human bodies, to foster student learning and counteract the contraction of hours granted to the teaching of anatomy, and the scarcity of bodies available for anatomical dissection [3, 26]. Computer-based learning covers a wide variety of devices, from tablets to smartphones, to computers, to the introduction of augmented and virtual reality, with a different degree of interaction between the student and the teacher and varying degree of usefulness reported in the literature. Indeed, there is no clear proof that computer-based learning is preferred by students or could entirely replace lectures, textbook use, or even dissection or prosection [17], but evidence suggests its possible role in supplementing traditional teaching methods [26].

As a consequence, the integration of the classical gross dissection with a supplemental virtual experience on a digital human cadaver hypothetically improving the learning of anatomy by medical students was tested. The introduction of a one life-size virtual dissection table with preinstalled whole body cross-sections of fresh frozen cadaver specimens permitted to the students to virtually manipulate structures in any plane or slice in cross-section, create three-dimensional reconstructions and add-on label to anatomical structures [29].

In the present randomised controlled didactical trial, almost all students (70%) belonging to the group who used the virtual dissection reported it was beneficial to their learning, making it a more engaging experience than studying textbooks and atlases, consistently to previous reports [8]. Coherently, during the time dedicated to learning (20 min), students effectively interacted with the display to actively process the material as verified by the personnel, hence avoiding the passive ‘watching’, especially of dynamic visualizations appearing self-explanatory, as previously described [5]. On the other hand, it was avoided asking participants to perform a specific activity during the learning phase, such as inferring the different perspectives or the movements of a structure, as it would over-stimulate them in active learning, fostering the process of understanding as previously described [5].

Overall, some difficulties have been reported both for students and teachers. Students had to overcome the learning curve to use the tool and complained table slowing, poor quality of images, especially during maximum magnifications, limits to the manipulations allowed by the software, and the restricted number of users simultaneously active on the device. The teachers have sometimes noticed some off-task behaviors of the students while using the virtual table, which however were quickly interrupted. Generally, the traditional gross dissection of a real body was judged superior to the potential virtual substitute. Anyhow, the preliminary usefulness of virtual dissection for subsequent application to traditional cadaveric dissection was recognized. The trial design prevented to investigate if medical students using the virtual dissection feel disadvantaged if they cannot attend dissection classes [3], as all the students performed a body dissection due to the planned experimental design. Moreover, it was not possible to make available on the virtual dissection provided to the students the computed-tomography scans specifically belonging to the dissected body, as other authors did [18]. However, it was believed that this was not a significant drawback. Indeed, the same authors reported that it would not be necessary to scan the same cadaver used during the dissecting session, given that a representative pre-installed data set was alternately made available [18], as in the present setting. This choice implied a clear advantage in cost and time savings without significantly affecting the learning experience.

It was found that medical students who applied to the virtual dissection aimed at learning anatomy were over three times more likely to report a positive outcome at the post-dissection test than those who applied to textbooks of topographical anatomy, controlling for all other factors in the model. Moreover, better results were noted for listing anatomical terms, as well as for 2D and 3D reporting of anatomical structures by students, who experienced the additional virtual dissection, even if the underpowering of analysis for these secondary outcomes impose wariness in interpreting the results. This matter is relevant, as the integration of the systematic 2D information learned in a 3D mental representation that synthesizes the different relationships between structures present in a specific anatomical district is of particular importance in clinical practice. Indeed, it is about the anatomical knowledge responding to the three-dimensionality of the human body with which the physician will have to interact in daily practice.

The present findings are consistent with Paech et al., who compared medical students applying to a conventional anatomy learning (dissection) to medical students adding the virtual to real dissection, although without randomisation of any student to any cohort [23]. They found that the latter performed better on the final test than the former, suggesting that simultaneous physical and virtual dissections provide unique conditions to study anatomy, allowing the achievements of higher scores [23].

Apart from the above-mentioned paper in literature, once excluded embryologic and dental issues, no other controlled nor randomised didactical studies evaluating the specific impact of virtual dissection as an addictive learning tool already integrated into the traditional cadaveric dissection of fresh frozen bodies were found.

Bork et al. compared the outcomes of using the AR Magic Mirror system and traditional radiology atlases versus the Anatomage during a two-semester gross anatomy course with first-year medical students and a follow-up elective course [6]. It was found that students improved subjectively assessed spatial understanding as well as their anatomical knowledge, with an advantage over traditional textbooks of both Anatomage and Magic Mirror. Moreover, for image questions, both the Anatomage and the theory subgroups showed an equivalent significant improvement between pre- and post-test. Overall, students reported that Anatomage is a valuable addition for enhancing dissection courses, albeit not as a full replacement [6].

Fyfe et al. replaced cadaver materials with Anatomage due to the increased student numbers and limited cadaver and/or prosections. Their cross-sectional study showed as the virtual dissection was promising, although requiring careful curriculum design and training to optimise its usefulness for learning [11].

Anand et al. performed a randomised cross-sectional prospective study on medical students comparing the virtual dissection table to traditional dissection [1]. They found no statistically significant difference among groups in the gain of knowledge, showing that virtual dissection table facilitates 3D visualization of structures and their relations being as good as traditional dissection [1].

Custer et al. investigated the use of virtual dissection by Anatomage in the education of imaging science students to assess their perceptions in learning imaging-based anatomy [8]. They reported that 96% of students felt that virtual dissection was a beneficial tool for learning anatomy [8].

Apart from the few above-mentioned full-text reported in the literature, we point out the significant presence of several congressional contributions on the subject, which—at the best of our knowledge—were not followed by the subsequent publication of a standard peer-reviewed paper, and hence was not included into the present paper.

More broadly and without a direct reference to the Anatomage, the results of some more studies useful for the discussion of the topic are reported.

Hisley et al. performed a randomised controlled study involving undergraduate first-year medical students, aimed to compare the physical dissection using an embalmed cadaver versus the digital dissection by 3D volume modeling of the whole body. Notably, the digital dissectors generated and manipulated 3D digital models. After six weeks, it was noted that students performed significantly better on questions presented as rotating models requiring spatial ordering or viewpoint determination responses, in contrast to requests for specific lexical feature identifications [13].

On the contrary, Donnelly et al. performed a cross-over study involved first-year medical students, aimed to investigate the use of a virtual human dissector in facilitating students’ ability to interpret cross-sectional images and understand the relationships between anatomical structures. The investigation was undertaken as self-directed learning activities using virtual computer-based dissector and prosections/models in the dissecting room. He noted no significant differences between the two groups at any tested stage [10].

Codd and Choudhury performed a controlled study involving second-year undergraduate medical students, aimed to evaluate the use of 3D virtual reality when compared with traditional anatomy teaching methods. A control group with no prior knowledge of forearm anatomy, a traditional methods group using dissection and textbooks, and a model group solely using e-resource were analysed. They concluded that learning-based on virtual anatomy could be used to complement traditional teaching methods effectively [7].

Metzler et al. performed a randomised controlled study involving undergraduate medical students, aimed to evaluate whether training on 3D presentation enhances the understanding of 2D images by randomisation to both the methods. The authors concluded that the correct interpretation of 2D imaging does not differ in students trained in either 3D or 2D [20].

They generally are in contrast to each other, being totally [1, 7, 8, 11, 23], partially [6, 13], or not at all [10, 20] supportive of the use of virtual dissection in teaching anatomy.

Results from the present study are hardly comparable to the previously published ones, due to (i) the different nature of the present experimental study, which is based on a randomised controlled design; (ii) the trial design, which included the same basic exposure to the real body dissection with the integration by an additional different learning tool (virtual dissection vs. classic textbook); (iii) the nature of handled bodies, which were fresh-frozen cadavers instead of embalmed ones or prosections.

In literature, also studies of lower quality according to the Oxford Scale for Evidence or weakly/not focused on the direct comparison between the study on textbook and use of virtual dissection showed inconsistent evidence on the usefulness of computer/mobile-based models in teaching anatomy [2]. Nevertheless, the integrative use of interactive media for anatomy teaching seems to be of added value, as it enhances independent learning, problem-solving, and provides flexibility in thought [27]. At the same time, it is unquestionable that cadaver-based learning by classic gross dissection is still a prerequisite for optimal training even with the use of informatics; however, they may resemble the real anatomical section, which still ranks highly [4, 26]. Namely, virtual dissection can play an important role in the acquisition of 3D anatomy knowledge, easily overcoming spatial difficulties encountered with traditional learning on textbooks, as it provides direct visualization of change throughout the viewpoints by dynamic visualizations, as well as permitting the unlimited go-ahead and get-back appraisal of anatomical relationships to enhance the teaching of complex areas including some that are difficult to display even by real dissection [5]. On this basis, it is believed that the virtual dissection cannot replace traditional ones, but could instead supplement it by offering students one more way to explore anatomy, both in class and on their own without requiring additional laboratory time allotment in a context of progressive curricular downsizing of training through anatomical dissection.

Nevertheless, there are some drawbacks to be accounted for when considering the introduction of virtual dissection into the gross anatomy curriculum. First, the lacking of scientific validation proven by peer-revision of information provided through informatics may represent an alarming issue, as most of anatomical software/apps have no medical expert involved during their development. Second, the cost to be faced for the purchase of a table for virtual dissection is of the order of tens of thousands of euros, all in a historical moment of contraction of the economic resources available. These points can constitute important drawbacks to the development of a truly integrated system for the teaching of anatomy and deserve to be tackled seriously in future studies.

The strengths of the present paper are the randomised controlled experimental design according to the CONSORT guidelines implemented to investigate in a focused fashion a debated issue concerning anatomical teaching. Moreover, the standardization of the methodology took into consideration Bloom's Taxonomy and in-depth statistical analysis.

However, the following limitations are present. First, the study is affected by small sample size and a low participation rate. These two aspects depend on the voluntary basis of students recruitment which fell in a period close to the exam session.

Second, the study population was restricted to second-year medical students. Whether these findings could be generalized to more senior ones needs to be determined. It has been shown in the literature that expertise could compensate for 3D reporting of anatomical structures, which are mainly improved in the group randomised to additional virtual dissection [5].

Third, students were not tested for their spatial ability, i.e., the aptitude for understanding a three-dimensional structure, which could interfere with the spatial learning process apart from the type of educational materials involved.

Fourth, the voluntary base of participation in the trial potentially leads to biases in various directions with respect to which students chose to attend the experiment. However, students were initially blinded to the experimental design to avoid attraction of people particularly interested in informatics, hence probably more responsive to interactive media. Moreover, students’ skills in informatics were not checked, but this aspect was considered not impacting on results as participants were recruited being not aware of the planned tasks.

Fifth, just one anatomical area was explored in this trial. The extension of the experiment to other anatomical regions could be of interest, deserving attention in further studies to determine the degree of generalisability referred to the anatomical topic.

Finally, the current results arise from a single-centre study, and the number of participants in the elective anatomy course was restrained. Therefore, further confirmations regarding external validity are required.

In conclusion, the present randomised controlled didactical trial showed that the combination of virtual to traditional gross dissection resulted in a significant improvement of second-year medical students’ learning outcomes.

The virtual dissection serves as a useful integrative tool in teaching and learning anatomy when subjected to validation by peer-review and used in conjunction with traditional gross dissection, which cannot be fully substituted by any virtual tool. It could be of help in maximizing the impact of practical experience, overcoming the contraction of economic resources, and the shortage of available bodies.

Nevertheless, further studies performed in multiple centres are needed to validate the results, to reduce further the experimental variance by increasing the sample size, to investigate additional independent variables of interest for adjusting the measured outcomes (e.g., personal details, pre-academic education, spatial ability, previous training with video games, etc.), and to examine the long-term impacts of this combined model on the learning process and the retention of anatomical concepts.

## Electronic supplementary material

Below is the link to the electronic supplementary material.

Supplementary file1 (DOCX 21 kb)

Exemplary multiple choice question from the pre-test (DOCX 16 kb)

Exemplary questions from the post-test (DOCX 25 kb)

Overview of subscores comparisons regarding the listing, the 2D and 3D reporting of anatomical structures. Text: experimental group randomised to the use of textbooks of topographical anatomy; VD: experimental group randomised to the virtual dissection; 2D: reporting of anatomical structures organized on a bidimensional plane; 3D: reporting of anatomical structures organized on a tridimensional volume. Dashed lines between medians identify the differences between groups. Percentages are referred to the proportion of correct answers provided (TIF 250 kb)
