# Supplementary material for: The additional role of virtual to traditional dissection in teaching anatomy: a randomised controlled trial
Source: Surg Radiol Anat. 2020 Sep 17;43(4):469–79. doi: 10.1007/s00276-020-02551-2 (PMC8021520; doi:10.1007/s00276-020-02551-2)
Supplement: Supplementary file 1 — Supplementary file1 (DOCX 21 kb) [file 276_2020_2551_MOESM1_ESM.docx]

**SURGICAL AND RADIOLOGIC ANATOMY**

**The additional role of virtual to traditional dissection in teaching anatomy. A randomised controlled trial.**

BOSCOLO-BERTO Rafael, TORTORELLA Cinzia, PORZIONATO Andrea, STECCO Carla, PICARDI Edgardo Enrico Edoardo, MACCHI Veronica, DE CARO Raffaele

Corresponding author: Prof. Veronica Macchi, MD, Institute of Human Anatomy, Department of Neurosciences, University of Padova, Via A. Gabelli 65, Padova 35127, Italy, E-mail: [veronica.macchi@unipd.it](mailto:veronica.macchi@unipd.it), Phone: 0039 049 8272300, Fax: 0039 049 8272319

**CONSORT checklist of information to include when reporting a randomised trial**

| **Section/Topic** | **Item number** | **Description** | **Page** |
| --- | --- | --- | --- |
| **Title** | 1a | Identification as a randomised trial in the title | 1 |
| **Abstract** | 1b | Mention of the design | 2 |
| **Introduction** |  |  |  |
| Background | 2a | Scientific background and explanation of rationale | 3-5 |
| Objectives | 2b | Objectives | 5 |
| **Background** |  |  |  |
| Trial design | 3a | Description of the design features including allocation ratio | 6 |
| Change from protocol | 3b | Not performed | Not applicable |
| Participants | 4a | Eligibility criteria for participants | 6 |
| Settings and location | 4b | Settings and locations where the data were collected | 6 |
| Interventions | 5 | Interventions | 7 |
| Outcomes | 6a | Definition of prespecified primary outcome measures | 5-6 |
| Changes to outcomes | 6b | Not performed | Not applicable |
| Sample size | 7a | Information on sample size | 6 |
| Interim analyses and stopping guidelines | 7b | Not performed | Not applicable |
| **Randomisation** |  |  |  |
| Sequence generation | 8a | Method used to generate the random allocation sequence | 6 |
| Sequence generation | 8b | Type of randomisation | 6 |
| Allocation concealment mechanism | 9 | Mechanism used to implement the random allocation sequence | 6 |
| Implementation | 10 | Who managed the allocation, enrolled participants, and assigned participants to the sequence of interventions | 6 |
| Blinding | 11a | Blinding details | 7 |
| Similarity of interventions | 11b | If relevant, description of the similarity of interventions | 6-7 |
| Statistical methods | 12a | Statistical methods used to compare groups | 9-10 |
| Additional analyses | 12b | Not performed | Not applicable |
| **Results** |  |  |  |
| Participant flow | 13a | Diagram | Figure 1 |
| Losses and exclusions | 13b | Number of participants excluded at each stage, with reasons | 10 |
| Recruitment | 14a | One-week trial | 7 |
| Trial end | 14b | One-week trial | 7 |
| Baseline data | 15 | A table showing baseline demographics | Table 1 |
| Numbers analysed | 16 | Number of participants included in each analysis | 10 |
| Outcomes and estimation | 17a | Results for each outcome | 10-12 |
| Binary outcomes | 17b | Presentation of both absolute and relative effect sizes | 11-12  Table 3 |
| Ancillary analyses | 18 | Not performed | Not applicable |
| Harms | 19 | Not applicable | Not applicable |
| **Discussion** |  |  |  |
| Limitations | 20 | Trial limitations | 20-21 |
| Generalisability | 21 | Generalisability and external validity | 20-21 |
| Interpretation | 22 | Interpretation consistent with results | 13-20 |
| **Other information** |  |  |  |
| Registration | 23 | Not registered | Not applicable |
| Protocol | 24 | Not registered | Not applicable |
| Funding | 25 | None | 1 |
