# Supplementary material for: The additional role of virtual to traditional dissection in teaching anatomy: a randomised controlled trial
Source: Surg Radiol Anat. 2020 Sep 17;43(4):469–79. doi: 10.1007/s00276-020-02551-2 (PMC8021520; doi:10.1007/s00276-020-02551-2)
Supplement: Supplementary file 2 — Exemplary multiple choice question from the pre-test (DOCX 16 kb) [file 276_2020_2551_MOESM2_ESM.docx]

**SURGICAL AND RADIOLOGIC ANATOMY**

**The additional role of virtual to traditional dissection in teaching anatomy. A randomised controlled trial.**

BOSCOLO-BERTO Rafael, TORTORELLA Cinzia, PORZIONATO Andrea, STECCO Carla, PICARDI Edgardo Enrico Edoardo, MACCHI Veronica, DE CARO Raffaele

Corresponding author: Prof. Veronica Macchi, MD, Institute of Human Anatomy, Department of Neurosciences, University of Padova, Via A. Gabelli 65, Padova 35127, Italy, E-mail: [veronica.macchi@unipd.it](mailto:veronica.macchi@unipd.it), Phone: 0039 049 8272300, Fax: 0039 049 8272319

**EXEMPLARY MULTIPLE CHOICE QUESTION FROM THE PRE-TEST**

**Only one answer (C) is correct**

***From which of the following bone structures does the flexor carpi radialis originate?***

A) ulnar tuberosity

B) lateral epicondyle of the humerus

C) medial epicondyle of the humerus

D) radial tuberosity

E) olecranon of ulna
