# Supplementary material for: The additional role of virtual to traditional dissection in teaching anatomy: a randomised controlled trial
Source: Surg Radiol Anat. 2020 Sep 17;43(4):469–79. doi: 10.1007/s00276-020-02551-2 (PMC8021520; doi:10.1007/s00276-020-02551-2)
Supplement: Supplementary file 3 — Exemplary questions from the post-test (DOCX 25 kb) [file 276_2020_2551_MOESM3_ESM.docx]

**SURGICAL AND RADIOLOGIC ANATOMY**

**The additional role of virtual to traditional dissection in teaching anatomy. A randomised controlled trial.**

BOSCOLO-BERTO Rafael, TORTORELLA Cinzia, PORZIONATO Andrea, STECCO Carla, PICARDI Edgardo Enrico Edoardo, MACCHI Veronica, DE CARO Raffaele

Corresponding author: Prof. Veronica Macchi, MD, Institute of Human Anatomy, Department of Neurosciences, University of Padova, Via A. Gabelli 65, Padova 35127, Italy, E-mail: [veronica.macchi@unipd.it](mailto:veronica.macchi@unipd.it), Phone: 0039 049 8272300, Fax: 0039 049 8272319

**EXEMPLARY QUESTIONS FROM THE POST-TEST**

**Bloom’s taxonomy – Knowledge domain**

**Students had to retrieve, and recall relevant knowledge from memory**

***List the names of anatomical structures (bones, muscles, vessels and nerves) in the forearm.***

**_____________________________________________________________________________**

**Bloom’s taxonomy – Comprehension domain**

**Students had to elucidate topographic relationships between anatomical structures**

***Name the bony structures and draw the anatomical structures (muscles, vessels and nerves) of the following proximal section of the left forearm.***

|  |
| --- |
